# Risk Factors for Death from Influenza A(H1N1)pdm09, State of São Paulo, Brazil, 2009

**DOI:** 10.1371/journal.pone.0118772

**Published:** 2015-03-16

**Authors:** Ana Freitas Ribeiro, Alessandra Cristina Guedes Pellini, Beatriz Yuko Kitagawa, Daniel Marques, Geraldine Madalosso, Gerrita de Cassia Nogueira Figueira, João Fred, Ricardo Kerti Mangabeira Albernaz, Telma Regina Marques Pinto Carvalhanas, Dirce Maria Trevisan Zanetta

**Affiliations:** 1 Epidemiological Surveillance Center, Disease Control Coordination, State of São Paulo Department of Health, São Paulo, Brazil; 2 Department of Epidemiology, School of Public Health, University of São Paulo, São Paulo, Brazil; Georgia State University, UNITED STATES

## Abstract

This case-control study aimed to assess the risk factors for death from influenza A(H1N1)pdm09 in patients with laboratory confirmation, who had severe acute respiratory illness-SARI and were hospitalized between June 28^th^ and August 29^th^ 2009, in the metropolitan regions of São Paulo and Campinas, Brazil. Medical charts of all the 193 patients who died (cases) and the 386 randomly selected patients who recovered (controls) were investigated in 177 hospitals. Household interviews were conducted with those who had survived and the closest relative of those who had died. 73.6% of cases and 38.1% of controls were at risk of developing influenza-related complications. The 18-to-59-year age group (OR = 2.31, 95%CI: 1.31–4.10 (reference up to 18 years of age)), presence of risk conditions for severity of influenza (OR = 1.99, 95%CI: 1.11–3.57, if one or OR = 6.05, 95%CI: 2.76–13.28, if more than one), obesity (OR = 2.73, 95%CI: 1.28–5.83), immunosuppression (OR = 3.43, 95%CI: 1.28–9.19), and search for previous care associated with the hospitalization (OR = 3.35, 95%CI: 1.75–6.40) were risk factors for death. Antiviral treatment performed within 72 hours of the onset of symptoms (OR = 0.17, 95%CI: 0.08–0.37, if within 48hours, and OR = 0.30, 95%CI: 0.11–0.81, if between 48 and 72 hours) was protective against death. The identification of high-risk patients and early treatment are important factors for reducing morbi-mortality from influenza.

## Introduction

In March and April 2009, health professionals warned the Mexican Ministry of Health about the increase in incidence of acute respiratory illness in young adults [1]. In April, a new viral subtype of influenza A(H1N1)pdm09, a combination of swine, avian and human virus, was identified in the United States and in samples originated from Mexico [2,3]. On April 25^th^, the World Health Organization-WHO declared that this event was a public health emergency and, on June 11^th^, it announced the influenza pandemic [4,5].

In Brazil, a new subtype of human influenza is included in the list of compulsory notification diseases since 2006, which is done in the Information System for Notifiable Diseases-SINAN. In May 2009, the first cases of Influenza-A (H1N1)pdm09 were confirmed in the country. With the beginning of the epidemics, the Secretariat of Health Surveillance elaborated a system of online reporting through the web application SINAN-flu to make the registration of cases easier and more opportune. After confirmation of transmission in the country in July, the guidelines for influenza surveillance gave priority for notification and investigation of cases of influenza-associated severe acute respiratory syndrome. During that year, 48,978 cases were confirmed and 2,051 deaths occurred, mainly in Southern and Southeastern regions, with mortality rates of 3.0 and 1.2 deaths per 100,000 inhabitants, respectively [6]. In São Paulo state, 12,002 cases were confirmed, of which 578 resulted in deaths, with a mortality rate of 1.4 per 100,000 inhabitants [7]. The epidemic initially spread through the metropolitan regions of São Paulo and Campinas, subsequently reaching the inner cities of the state, with a peak of transmission in the first week of August [8].

In the United States, between April and May 2009, 60% of cases occurred in children and adolescents, and only 5% in those aged 51 years and over[9]. In Mexico, 87% of deaths and 71% of cases were concentrated in those between five and 59 years, whereas the seasonal influenza from 2005 to 2008 totaled 17% and 32%, respectively [10]. In 2009, hospitalization rate varied from 2.9 per 100,000 inhabitants in Japan to 24.5 in Argentina, while this rate was 8.8 in Brazil. The presence of co-morbidity was frequent among hospitalized cases, from 27% in the United States to 79% in Brazil [11]. Chronic pulmonary diseases were the main pre-existing diseases among children and adults hospitalized in the United States [12].

Being aged 18 or over, antiviral therapy beginning more than 48 hours after the onset of symptoms [13] and the presence of obesity (BMI>40) or chronic liver disease [14] were risk factors for greater severity in patients hospitalized due to influenza A(H1N1)pdm09, admitted to Intensive Care Units-ICU. A study performed with patients admitted to ICUs in Spain showed that cancer, immunodeficiency and morbid obesity were associated with death from influenza in adults [15]. Although several studies have sought to identify factors associated with disease severity, few have analyzed the risk factors for death.

The present study aimed to identify risk factors for death from influenza A(H1N1)pdm09 in hospitalized patients with associated severe acute respiratory illness-SARI. This knowledge is important to support future interventions during pandemics, taking into account the organization of health services and selection of priority groups for vaccination.

## Methods

The state of São Paulo is the most populous in Brazil, with an estimated population of 41,384,089 inhabitants in 2009 (21.6% of the Brazilian population). The metropolitan region of São Paulo includes 39 cities, and that of Campinas, 42 cities, corresponding to 47.8% and 9.6% of the state’s population, respectively.

During the epidemic in 2009, 7,400 patients who lived in the metropolitan regions of São Paulo and Campinas were notified and confirmed as having influenza A(H1N1)pdm2009, of whom 4,066 were associated with SARI, resulting in 328 deaths.

**Fig 1:**
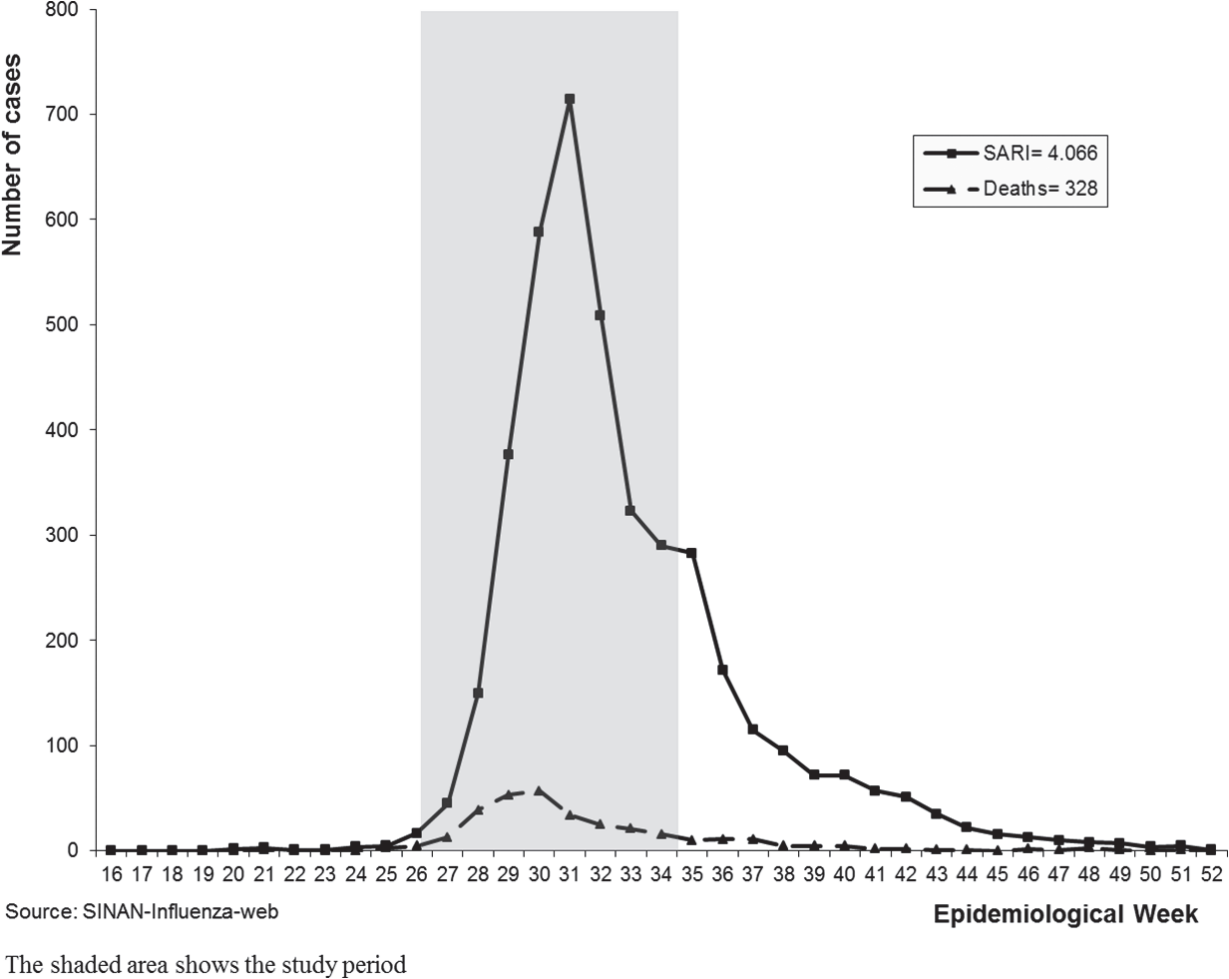
Number of confirmed cases and deaths from Influenza A(H1N1)pdm 09 among hospitalized SARI patients, according to the epidemiological week of onset of symptoms, metropolitan regions of São Paulo and Campinas, Brazil 2009.

A case-control study was performed with patients who had been hospitalized for more than 24 hours, with a confirmed infection of Influenza A(H1N1)pdm09 and SARI, between June 28^th^ and August 29^th^ 2009. The study period included the majority of disease confirmations and deaths from this epidemic in 2009, as shown in Fig. 1.

The patients were identified in the SINAN database, using the following variables for selecting the cases and controls: RT PCR (positive for influenza A(H1N1)pdm2009), final classification (confirmed), criteria (laboratory), evolution (recovered or death), hospitalization (yes), date of hospitalization (28/06/2009 to 29/08/2009), municipalities of residence and hospitals (metropolitan areas of Sao Paulo and Campinas).

At the time the patients were selected for the study, the SINAN included 193 reported and confirmed deaths of patients with influenza A(H1N1)pdm2009 and SARI, all of which were assessed in this study (cases). For each case of death, two control patients were selected among the 1988 patients hospitalized during the study period with reported and confirmed influenza A(H1N1)pdm2009 and SARI who recovered. The controls were time-paired randomly selected from those hospitalized in the same epidemiological week (EW) or, if necessary, one week later than the date of hospitalization of cases to prevent possible differences in opportunity of treatment and clinical management protocol.

Data of the study were collected by trained health professionals using two standardized questionnaires. The hospital questionnaire collected information from medical charts, evaluated in 177 hospitals were patients were hospitalized. Of these, 56.5% belonged to the Unified Health System-SUS and 43.5% were private hospitals. The following data were collected: previous pathological history, occurrence of pregnancy, health care, symptomatology during admission, laboratory and radiological tests, need for ICU admission, treatment, complications and disease progression. The International Classification of Diseases, 10^th^ revision-ICD-10, was used for coding comorbidities indicated in the medical charts. The Ministry of Health provided the antiviral drug known as Oseltamivir for treatment during the pandemic. The protocol for clinical management established that the treatment was for cases hospitalized with SARI and ambulatory patients with risk conditions for severity. Treatment should be started within 48hours after symptom onset.

All patients studied had laboratory confirmations of influenza A(H1N1)pdm09 from respiratory secretion samples analyzed using the RT-PCR method, performed by the Adolfo Lutz Institute [16]. The definition of SARI included: fever associated with cough and dyspnea; pneumonia; respiratory insufficiency; tachypnea; radiological changes compatible with pneumonia; oxygen therapy or mechanical ventilation. SARI was confirmed in all cases and controls selected for the study by data their on medical charts.

Household interviews were conducted in 161 cases (83.4%), with 14 refusals (7.3%) and 18 (9.3%) addresses not found. A total of 337 (85.1%) controls were interviewed, with 6 (1.5%) refusals and 43 (11.1%) addresses not found. The household questionnaire included socio-demographic data, history of previous care related to the episode that led to hospitalization and presence of influenza vaccination. Occupations were grouped according to the occupational pyramid of influenza risk [17]. For cases, interviews were conducted with the closest family members; for controls, with the patients themselves, excluding children, whose parents/legal guardians responded for them.

After data collection, questionnaires were assessed by supervisors to verify the consistency of information and the fulfillment of criteria to include patients in this study. Hospital and household questionnaires were input to the Sphynx database, version 5.

The present study began in order to assist with decisions on surveillance and control actions. The use of data was approved by the Research Ethics Committee, School of Public Health, University of São Paulo-FSP-USP (protocol 2283, OF.COEP/312/11). Data collection was performed in accordance with the recommendations of the National Health Council for Human Research, including the signing of an informed consent form by patients, family members or legal guardians.

Clinical and demographic characteristics are shown as median and inter-quartile intervals or percentages, and comparisons were made using the Mann-Whitney U or chi-square tests, as required. Odds ratio-OR and 95% confidence intervals-95%CI were calculated to assess the factors associated with death. Variables with p-value < 0.20 in the univariate analysis and adjustment variables were selected for the multiple logistic regression, which used forward stepwise strategy. The model including all patients assessed the following dichotomous categorical variables: health insurance, previous care, vaccination against influenza in 2009, sex (reference category = male); and the following risk conditions: diabetes mellitus, obesity, chronic kidney disease, chronic liver disease, immunosuppression, chronic cardiovascular disease, chronic neurological disease and delay in development, using the absence of a condition as a reference category. The model also assessed: age group [<18 years (reference), 18 to 59 years, ≥ 60 years], use of antiviral therapy [no use (reference category), ≤ 48 hours after the onset of symptoms, > 48 and ≤ 72 hours, > 72 hours], high-risk individuals or those with any of the risk conditions listed above adapted from the Centers for Disease Control and Prevention-CDC [18] [no condition (reference category), one condition, and two or more conditions], and time between onset of symptoms and admission in days as the continuous variable. The model was controlled for epidemiologic week of admission at hospital (categorized as EW26-28 (reference), EW29-31 and EW31-34), and income. A second model was developed with patients aged 18 years and over, which also assessed level of education [without formal education or incomplete primary education, complete primary education, complete secondary education, complete higher education (reference category)] and occupational risk [very high and high, average and low (reference category)]. Patients aged 18 to 22 years with incomplete higher education were included in reference category of education. For the modelling, patients with no occupation were included in the reference category of occupational risk. Significance of variables was assessed by Wald test and adjustment of models by Hosmer-Lemeshow test.

The analyses were performed using SPSS software, version 17.0. P-values < 0.05 were considered to be significant.

## Results

Table 1 presents the socioeconomic characteristics of patients and information associated with the disease obtained from the household interviews. Among patients aged 18 years and over, there was a higher risk of death for those without formal education or with incomplete primary education, OR 2.33(95% CI 1.13–4.89) and complete primary education, OR 3.06 (95%CI 1.41–6.82), when compared to those with higher education. Distribution of ethnic groups, family income and history of smoking were not associated with death. The history of previous vaccination against seasonal influenza in 2009 showed protection against death. Health professionals, considered to be at a high risk of developing influenza, showed protection against death, OR 0.15(95%CI 0.02–0.61). Patients who had sought care prior to hospitalization had a risk of death 3.70 times (95%CI 2.25–6.30) that of patients without previous care, with a predominance of emergency care (45.34%).

**Table 1:** General and socio-demographic characteristics of patients hospitalized due to influenza A(H1N1)pdm09 associated with Severe Acute Respiratory Illness, who died (case) or recovered (control), metropolitan regions of São Paulo and Campinas, state of São Paulo, Brazil, 2009.

		**N (%) CASE** ^**a**^	**N (%) CONTROL** ^**a**^	
**CHARACTERISTICS**		**(n = 159)**	**(n = 335)**	**OR** ^**b**^ **(95% CI)**
Ethnicity^c^	White	113 (71.1)	219 (65.4)	1
	Black/Mixed	43 (27.0)	106 (31.6)	0.79 (0.51–1.20)
Private health insurance		67 (42.1)	166 (49.6)	0.74 (0.51–1.08)
Previous care		138 (86.8)	214 (63.9)	3.71 (2.25–6.30)
2009 influenza vaccine^d^		13 (8.2)	53 (15.8)	0.51 (0.26–0.95)
Household income^e^	Up to 02 MW	60 (37.7)	138 (41.2)	1.13 (0.58–2.26)
	02 to 04 MW	54 (34.0)	87 (26.0)	1.61 (0.82–3.27)
	04 to 08 MW	27 (17.0)	61 (18.2)	1.15 (0.54–2.48)
	> 08 MW	15 (9.4)	39 (11.6)	1
**CHARACTERISTICS OF PATIENTS**		**N (%) CASE** ^**a**^	**N (%) CONTROL** ^a^	**OR** ^**b**^ **(95% CI)**
**AGED ≥ 18 YEARS**		**(n = 131)**	**(n = 192)**	
Level of education^f^				
Without formal education/Incomplete primary education		35 (26.7)	43 (22.3)	2.33 (1.13–4.89)
Complete primary education		28 (21.4)	26 (13.5)	3.06 (1.41–6.82)
Complete secondary education		52 (39.7)	77 (40.1)	1.90 (0.98–3.79)
Complete higher education		16 (12.2)	46 (24.0)	1
Smoking ^g^		23 (17.6)	41 (21.4)	0.78 (0.44–1.38)
Occupation		85 (64.9)	138 (71.9)	0.72 (0.45–1.17)
Occupational risk	Very high and high^h^	2 (2.4)	21 (15.6)	0.15 (0.02–0.61)
	Average^i^	52 (61.2)	65 (48.2)	1.26 (0.70–2.27)
	Low^j^	31 (36.5)	49 (36.3)	1

^a^Data collected from household interviews

^b^ Unadjusted OR

^c^ 2 controls ignored; 3 (1.9%) cases and 8(2.4%) controls were Asian/Indigenous

^d^12 cases and 3 controls ignored

^e^3 cases and 10 controls ignored, MW minimum wage (R$ 465.00 or US$ 236.34 at the time of this study)

^f^1 cases ignored; complete higher education includes 16 patients aged 18 to 22 years with incomplete higher education (2 cases and 14 controls)

^g^Smoked at the time of hospitalization

^h^Physicians, nurses, dentists, other health professionals and supporting professionals in health services

^i^Education, business, service and administrative professionals with close contact with the population

^j^Management professionals and other university and technical professionals without close contact with the population.

Table 2 presents the distribution of cases and controls according to variables assessed in the hospital investigation. Median age of cases was higher than that of controls (p<0.001) and the 18-to-59-year age group had an OR for death of 4.03(95%CI 2.62–6.31), compared to those aged less than 18 years. Elderly individuals were also at a significant risk of death.

**Table 2:** Demographic and clinical variables of patients hospitalized due to influenza A(H1N1)pdm09 associated with Severe Acute Respiratory Illness, who died (case) or recovered (control), metropolitan regions of São Paulo and Campinas, state of São Paulo, Brazil, 2009.

		**N (%) CASE** ^**a**^	**N (%) CONTROL** ^**a**^	
**CHARACTERISTICS**		**(n = 193)**	**(n = 386)**	**OR** ^**b**^ **(95% CI)**
Age group (years)	< 18	31 (16.1)	168 (43.5)	1
	18–59	152 (78.8)	204 (52.8)	4.03 (2.62–6.31)
	60 and more	10 (5.2)	14 (3.6)	3.84 (1.52–9.50)
Age, median (IQR)^c^		32.97 (22.89–46.32)	20.88 (4.99–34.32)	*p*<0.001
Sex, female		118 (61.1)	217 (56.2)	1.22 (0.86–1.75)
Risk conditions	None	51 (26.4)	239 (61.9)	1
	At least one	142 (73.6)	147 (38.1)	4.49 (3.09–6.60)
	Presence of one	79 (40.9)	117 (30.3)	3.16(2.09–4.80)
	> one	62 (32.6)	28 (7.3)	10.29 (6.04–17.85)
Obesity^d^		58 (30.1)	20 (5.2)	7.83 (4.58–13.76)
Pregnancy^e^		23 (28.8)	48 (36.4)	0.71 (0.38–1.29)
Asthma		14 (7.3)	40 (10.4)	0.68 (0.35–1.26)
Chronic pulmonary disease^f^		15 (7.8)	16 (4.1)	1.95 (0.93–4.07)
Diabetes mellitus		35 (18.1)	12 (3.1)	6.88 (3.53–14.09)
Immunosuppression^g^		25 (13.0)	15 (3.9)	3.67 (1.89–7. 30)
Chronic cardiovascular disease^h^		29 (15.0)	14 (3.6)	4.68 (2.43–9.34)
Chronic kidney disease		9 (4.7)	5 (1.3)	3.71 (1.23–12.40)
Chronic liver disease		5 (2.6)	1 (0.3)	10.24 (1.4–244.2)
Chronic neurological disease/Delay in development^i^		19 (9.8)	5 (1.3)	8.29 (3.17–25.25)
Hemoglobinopathies		2 (1.0)	4 (1.0)	1.00 (0.13–5.69)
Antiviral treatment^j^				
No use		59 (30.6)	87 (22.5)	1
Yes (use at any moment)		133 (68.9)	295 (76.4)	0.67 (0.45–0.98)
≤ 48 hours after the onset of symptoms		18 (9.3)	137 (35.5)	0.19 (0.11–0.35)
> 48 and ≤ 72 hours after the onset of symptoms		10 (5.2)	42 (10.9)	0.35 (0.16–0.74)
> 72 hours after the onset of symptoms		105 (54.4)	116 (30.1)	1.33 (0.87–2.04)
**Time/Days, median (IQR)** ^**c**^		**CASE**	**CONTROL**	**p-value**
First symptoms until hospitalization		5 (3–6)	2 (1–5)	<0.001
Hospitalization until discharge/death		7 (2–15)	4 (2–7)	<0.001
Onset of symptoms until the beginning use of antiviral treatment		6 (4–8)	3 (2–5)	<0.001
Hospitalization until the beginning of antiviral treatment		1 (0–2)	0 (0–1)	<0.001

^a^ Data collected from hospital records;

^b^ Unadjusted OR

^c^ IQR: Inter-quartile range. Minimum and maximal ages were 0.13 and 84.7 years of age for cases and 0.04 and 85.8 years of age for controls

^d^Recorded in medical charts;

^e^ The denominator of proportions are women of reproductive age (10 to 49 years), cases (80) and controls (132)

^f^Chronic pneumonia, chronic obstructive pulmonary disease, cystic fibrosis, bronchiectasis;

^g^Malignant neoplasm, autoimmune disease, use of immunosuppressant (corticosteroids/others), organ transplants and HIV/AIDS;

^h^Chronic cardiovascular disease, heart failure, peripheral vascular disease, cardiac arrhythmia, congenital disease, ischemic disease;

^i^Neurological diseases, delay in development, muscular dystrophy and Down’s syndrome;

^j^1 case and 4 controls ignored.

There were no differences in sex distribution in either group. With regard to risk conditions for severity, 73.6% of cases and 38.1% of controls reported having at least one risk factor, with an OR of 4.49 (95%CI 3.09–6.60). When individuals with more than one of these conditions were assessed, the risk of death increased, OR 10.29 (95%CI 6.04–17.85). The following medical conditions were risk factors for death: obesity, diabetes mellitus, chronic cardiovascular diseases, chronic kidney diseases, neurological diseases and delay in development, chronic liver diseases and immunosuppressive diseases. The immunosuppression-related diseases were: neoplasms (n = 18), HIV/AIDS (n = 8), organ transplants (n = 6), autoimmune diseases (n = 5) and use of immunosuppressants (n = 3).

Of the 212 women of reproductive age (10 to 49 years) who were studied, 33.5% were pregnant and there were no differences in the frequency of pregnancy in either group.

The use of antiviral treatment during hospitalization was a protective factor against death, with an OR of 0.67 (95%CI 0.45–0.98). When time between onset of symptoms and beginning of antiviral treatment was assessed, there was an 81% protection among individuals who received treatment within the first 48 hours, with an OR of 0.19 (95%CI 0.11–0.35), and 65% between 48 and 72 hours, with an OR of 0.35 (95%CI 0.16–0.74). After 72 hours, there was no significant protection. Median of days between date of onset of symptoms and date of hospitalization was 5.0 among cases and 2.0 among controls (p<0.001). Among patients with chronic pulmonary disease, median was 4.0 days among cases and 2.0 days among controls; among those with asthma, 4.5 days and 2.0 days; and among pregnant women, 4.0 days and 2.0 days, respectively. With regard to health professionals, the median between the date of onset of symptoms and that of hospitalization was three days for both cases and controls. Median between date of hospitalization and of death or hospital discharge was seven days among cases and four days among controls (Table 2).

According to medical charts, fever and cough occurred in 87.8% of cases and 79.3% of controls; and the proportions of dyspnea were 87% and 62.7%, respectively. The definition of cases of Severe Acute Respiratory Syndrome –SARS (fever+cough+dyspnea) based on information obtained from medical charts occurred in 68.9% of cases and 56.5% of controls. Vomit and diarrhea were present in 8% of cases and 18% of controls. Treatment with antibiotics was used in 95.8% of cases and 73.1% of controls. The mean number of antibiotics used was 4.0 and 1.8 respectively. Among cases, there was a higher proportion of hospitalizations in intensive care units (83.4%x16.8%), use of mechanical ventilation (94.8%x10.8%) and dialysis (25.0%x2.3%). The proportion of complications was higher among cases than controls, with an emphasis on: respiratory distress syndrome (64.7%vs4.9%), shock (69.4%vs1.0%), sepsis (63.2%vs2.3%) and kidney complications (44.6%vs1.3%), respectively.

Table 3 shows the laboratory findings of entry tests of patients in the hospital. Cases had lower numbers of leukocytes and platelets and higher values of creatine phosphokinase-CPK, lactic dehydrogenase-LDH, glutamic-oxaloacetic transaminase, glutamic-pyruvic transaminase, urea and creatinine than controls, with statistical significance.

**Table 3:** Laboratory tests of patients hospitalized due to influenza A(H1N1)pdm09 associated with Severe Acute Respiratory Illness, who died (case) or recovered (control), metropolitan regions of São Paulo and Campinas, state of São Paulo, Brazil, 2009.

		**CASE**		**CONTROL**	
**LABORATORY TESTS** ^**a**^	**N**	**Median (IQR)** ^**b**^	**N**	**Median (IQR)** ^**b**^	**p-value**
Hemoglobin	182	12.75 (10.8–12.8)	305	12.6 (11.3–14)	0.262
Hematocrit	181	38.6 (32.6–42)	293	37.9 (34–41.7)	0.974
Leukocytes	182	6,350 (4,075–9,812.5)	301	7,550 (5,395–10,500)	0.002
Platelets	179	166,000 (123,000–230,000)	292	228,500 (176,000–303,000)	<0.001
Creatine phosphokinase – CPK	67	350 (111–845)	35	92 (54–217)	<0.001
Lactic dehydrogenase – LDH	60	881.5 (483–1,907)	53	422 (255–551.50)	<0.001
Glutamic-oxaloacetic transaminase—GOT	118	81 (38–133.5)	71	31 (22–53)	<0.001
Glutamic-pyruvic transaminase – GPT	117	43.1 (29.5–74)	70	27.5 (17–41.5)	<0.001
Urea	175	32 (23–49)	165	25 (15.5–34)	<0.001
Creatinine	175	0.89 (0.6–1.3)	170	0.7 (0.5–0.9)	0.001

^a^First hospitalization blood test

^b^IQR: Inter-quartile range.

In sub-group analysis, previous conditions of risk for severity and obesity were risk factors for death in univariate analysis for children younger than 18 years and women of reproductive age (10 to 49 years). Among children, presence of immunosuppression, chronic cardiovascular disease and neurological disease/delay in development were also risk factors, as well as diabetes mellitus among women. Treatment with antiviral administered within 72 hours after the onset of symptoms was a protective factor for women of reproductive age.

Tables 4 and 5 show variables associated with death in the final models of multiple logistic regression, in all patients studied and in sub-group analysis of adults (aged 18 years and over). In patients in general, risk factors for death were: being aged between 18 and 59 years (reference: until 18 years), having one or more risk conditions for the severity of the disease, obesity, immunosuppression and having sought care prior to hospitalization. Although chronic neurological diseases/delay in development and chronic liver disease were highly associated with death, these variables were excluded from the final model because they had estimates with large confidence intervals. Private health insurance and antiviral treatment administered in the first 72 hours after the onset of symptoms were protective factors against death.

**Table 4:** Risk factors for death from influenza A(H1N1)pdm09 associated with Severe Acute Respiratory Illness, metropolitan regions of São Paulo and Campinas, state of São Paulo, Brazil, 2009.

**CHARACTERISTICS**		**ADJUSTED OR** ^a^ **(95% CI)**
Age group (years)	< 18	1
	18–59	2.31 (1.31–4.10)
	60 and more	1.44(0.39–5.39)
Risk conditions^b^	None	1
	Presence of one	1.99 (1.11–3.57)
	> one	6.05 (2.76–13.28)
Obesity^c^		2.73 (1.28–5.83)
Immunosuppression^c^		3.43 (1.28–9.19)
Previous care^c^		3.35 (1.75–6.40)
Antiviral treatment	No use	1
	≤ 48 hours after the onset of symptoms	0.17 (0.08–0.37)
	> 48 and ≤ 72 hours after the onset of symptoms	0.30 (0.11–0.81)
	> 72 hours after the onset of symptoms	1.10 (0.62–1.95)

^a^ significant variables in the final multiple logistic regression model. Hosmer-Lemeshow test = 0.069

^b^ risk conditions for severity of the disease

^c^ Reference category (absence).

**Table 5:** Risk factors for death from influenza A(H1N1)pdm09 associated with Severe Acute Respiratory Illness in adults ^a^, metropolitan regions of São Paulo and Campinas, state of São Paulo, Brazil, 2009.

**CHARACTERISTICS**		**ADJUSTED OR** ^b^ **(95% CI)**
Risk conditions ^c^	None	1
	Presence of one	1.70 (0.86–3.37)
	> one	4.78 (1.95–11.71)
Obesity^d^		3.06 (1.34–7.00)
Occupational risk^e^	Low risk	1
	Average risk	0.85 (0.46–1.59)
	High risk	0.16 (0.03–0.86)
Previous care^d^		3.84 (1.70–8.70)
Antiviral treatment	No use	1
	≤ 48 hours after the onset of symptoms	0.12 (0.04–0.34)
	> 48 and ≤ 72 hours after the onset of symptoms	0.37 (0.13–1.07)
	> 72 hours after the onset of symptoms	1.06 (0.52–2.16)

^a^≥ 18 years;

^b^significant variables in the final multiple logistic regression model. Hosmer-Lemeshow test = 0.854

^c^ risk conditions for severity of the disease

^d^Reference category (absence)

^e^ grouped according to occupational pyramid of influenza risk. Low risk included those with no occupation.

Among adults aged 18 years and over, the risk factors for death were having more than one risk factor for severity of the disease, obesity and care prior to hospitalization. The protective factors were: antiviral drug administered in the first 48 hours after the onset of symptoms and being a health professional or health unit worker.

Sensitivity analysis was performed to assess the severity of patients admitted during the study period. The proportion of ICU admissions and use of mechanical ventilation was estimated for epidemiological week of hospitalization, categorized as EW 26 to 28, EW 29 to 31 and EW 32 to 34. The results showed higher proportions of ICU admissions (50.0% x 16.8% x 7.7%) and mechanical ventilation (45.4% x 10.8% x 2.6%) in the three first weeks, among the controls. The cases showed high proportions in all periods analyzed: 89.7% x 84.0% x 76.9% for need of ICU and 89.7% x 97.6% x 89.7% for need of mechanical ventilation, in the 3 periods, respectively. The opportunity for antiviral treatment showed lower proportion of treatment in the first period of the study, in both cases (37.9% x 74.4% x 74.3%) and controls (59.1% x 74.1% x 89.7%). Sensitivity analysis to assess the opportunity for treatment of children and adults showed similar proportions for the groups: 73.9% and 74%, respectively.

## Discussion

In the present study, risk factors for death were analyzed in patients infected with influenza A(H1N1)pdm09 and hospitalized with SARI. There was an increased risk of death in patients aged 18 to 59 years; those with obesity, immunosuppression, neurological and developmental diseases; or those who had received care prior to hospitalization. Additionally, presence of at least one risk factor for aggravation of the disease was also associated with occurrence of death. Use of antiviral drugs, especially when administered in the first 72 hours after onset of symptoms was protective factor against death.

The design of this case-control study, including all deaths reported and confirmed in the region during the study period and the time-paired random selection of controls among patients who survived after hospitalization with SARI, with hospital and household data collection, enabled the analysis of the factors associated with death to be extended, including socioeconomic variables, access to health services, and data on hospitalizations.

Few studies have assessed risk factors for death in patients with SARI [15,19]. Some studies have analyzed risk factors for severity of the disease and deaths, comparing patients hospitalized in ICUs and in wards [13,14,20–23], whereas one study used the rate of mortality from influenza A(H1N1)pdm09 in two epidemic waves [24]. A study conducted in Australia analyzed risk factors for hospitalization due to influenza, comparing cases with controls without infection in the community [25]. Other studies have analyzed epidemiological surveillance data, including mortality rates and comparisons among deaths, hospitalizations, treatment and other factors [9,10,12,26–35]. Some of the factors associated with death identified in this study have been described by other authors, such as the presence of comorbidities [31,35], age≥18years [13], obesity [15,19,20], neurological and developmental diseases [23,24,31] and immunosuppression [15,21,24,34]. Children showed lower risk of death, despite a similar proportion of treatment than adults. A similar result was observed in a study of systematic review and meta-analysis, where the risk of death was 0.28 (95% CI 0.19–0.141), when compared to young adults [36]. In contrast, other factors associated with severity of infection that have been reported by different researchers, such as diabetes [19,34]; other chronic pulmonary diseases, excluding asthma and chronic obstructive pulmonary disease [20]; chronic pulmonary disease, including asthma [31]; and chronic cardiovascular disease [21,24,34], were not associated with mortality in the present study. The population assessed, which was restricted to hospitalized patients with severe influenza could at least partly explain these differences. Diabetes, chronic cardiovascular and kidney disease, associated with death in univariate analysis, did not remain risk factors after multiple analysis. The design of this study, with data collected on medical charts and household interviews, allowed for adjustments in the analysis that can also explain part of these differences.

Deaths occurred mainly in adults aged 18 to 59 years, with a small proportion of cases and controls among elderly individuals. A possible preexisting immunity against the new influenza A(H1N1)pdm09 virus in this age group has been suggested, probably due to previous exposure, through other infections or vaccinations, to an influenza A(H1N1) virus which is genetically and antigenically more associated with the new virus than contemporary strains of seasonal H1N1 viruses [37].

All patients in the present study, whether cases or controls, had a laboratory-confirmed infection of influenza A/H1N1pdm09, which was not similar to the viruses present in the 2009 trivalent influenza vaccine in the Southern hemisphere [A/Brisbane/59/2007(H1N1), A/Brisbane/10/2007 (H3N2) and B/Florida/4/2006] [38].

It is possible that the probability of being hospitalized varied throughout the study. Treatment was available for hospitalized patients and for ambulatory patients with risk conditions for severity. This could have influenced hospitalization, but not much in our study population, consisting of patients with SARI. During the pandemic, criteria for hospitalization changed as well as the availability of treatment, especially in the first 3 weeks of the epidemic, as shown in sensitivity analysis, confirming the need for controlling period of hospitalization in the study.

The socioeconomic profile was similar in both groups assessed, with no difference in income or having private health insurance. There was greater protection against death among patients with an occupation with higher risk of exposure to infection, such as health professionals. A greater opportunity of access to treatment among these workers could explain such findings, as the period between the date of onset of symptoms and the date of admission was shorter in this group. The low proportion of deaths among patients categorized as having a higher risk of exposure has also been observed in other studies [30,33].

Pregnancy was not a risk factor for death in this study. In contrast, frequency of pregnant women among those of reproductive age hospitalized with severe manifestation of influenza A(H1N1)pdm09 was high, suggesting that pregnancy could be associated with development of SARI. An association between pregnancy and use of mechanical ventilation during hospitalization due to influenza A(H1N1)pdm2009 [25] and a higher admission rate were reported when compared to the general population [32]. In 2009, in the state of São Paulo, 27.4% of all confirmed cases of influenza A(H1N1)pdm09 in women of reproductive age (10 to 49 years), hospitalized and recorded in the SINAN database, were found in pregnant women. In Brazil, only 4.27% of women of reproductive age had live newborns in the reference year according to the 2010 Census [39].

Patients who died presented important laboratory changes when compared to controls, with lower levels of leukocytes and platelets and an increase in CPK, LDH, urea and creatinine. Studies revealed a similar trend for the laboratory results of leukocytes [23], CPK [26,27,30], LDH [26], platelets [22] and creatinine [27,30,34].

Although most patients who died received antiviral treatment, only 9.3% were treated in the first 48 hours. Early treatment was a protective factor against death, especially when provided in the first 72 hours after onset of symptoms. Other studies have also shown a more favorable prognostic with antiviral treatment [13,21,23,27,28,29,34].

History of care prior to hospitalization was a risk factor for death from influenza A(H1N1)pdm09, which could reflect the difficulty in accessing health services. Emergency services were primarily sought for care prior to hospitalization, which could have contributed to greater delay in diagnosis and beginning of treatment. A study conducted in Mexico showed a similar result with regard to delay in hospitalization [29].

In one study, which assessed the overall standardized mortality rate from influenza A(H1N1)pdm09, the mortality among patients with preexisting chronic respiratory diseases was lower than those with other chronic diseases^24^. There might have been problems with recording information about previous diseases in the medical charts in the presence of acute pulmonary disease, especially among those with chronic pulmonary disease.

The present study had some limitations. Underreporting of SARI by health professionals is likely to have occurred, as reporting this disease was not compulsory in Brazil before the pandemic. However, during the pandemic, professionals were more sensitive to reporting it. The system for notification, which was mandatory during the epidemic, was facilitated to allow for rapid notifications. The availability of samples from autopsies of cases (deaths) could have resulted in greater access to the laboratory diagnosis of this infection. Household data collection could also have caused information bias, as data on cases were collected through interviews with family members, while data on controls were obtained from the patients themselves, when they were adults. The proportion of refusals of household interviews was higher among cases (7.3%) than controls (1.6%), p = 0.001. Another possible limitation was memory bias, as a result of the time between the hospitalization and interview. However, the information analyzed from household interviews was about socio-demographic characteristics, which is less vulnerable to information and memory bias. Data on preexisting diseases were obtained from medical charts in hospitals, where they are completed at the moment of hospitalization, in the presence of the patients. The quality of completion of medical charts can differ between hospitals. Nonetheless, information about the presence of chronic diseases, clinical management of patients, laboratory results, radiological tests and treatment was available in these charts. The difficulties encountered during data collection were minimized by the use of a standardized questionnaire.

In conclusion, the risk conditions for the severity of the disease, particularly obesity, immunosuppression and neurological/developmental diseases, were also important risk factors for death, emphasizing the need for good vaccination coverage during influenza vaccination campaigns in all patients with chronic diseases belonging to more vulnerable groups. Another relevant result was the antiviral treatment within the first 72 hours of onset of symptoms as a protective factor, also emphasizing the need to warn the population about the importance of seeking early medical care when an influenza-like illness appears, especially among high-risk groups and those with signs of disease aggravation. Training of health professionals, especially physicians, is required for adequate clinical management of patients and early antiviral treatment.
